# Research of visual attention networks in deaf individuals: a systematic review

**DOI:** 10.3389/fpsyg.2024.1369941

**Published:** 2024-05-09

**Authors:** Nahuel Gioiosa Maurno, Jessica Phillips-Silver, María Teresa Daza González

**Affiliations:** ^1^Department of Psychology, University of Almería, Almería, Spain; ^2^CIBIS Research Center, University of Almería, Almería, Spain; ^3^Growing Brains, Washington, DC, United States

**Keywords:** deaf, children, attention, hearing, orienting, review methodology, investigation

## Abstract

The impact of deafness on visual attention has been widely discussed in previous research. It has been noted that deficiencies and strengths of previous research can be attributed to temporal or spatial aspects of attention, as well as variations in development and clinical characteristics. Visual attention is categorized into three networks: orienting (exogenous and endogenous), alerting (phasic and tonic), and executive control. This study aims to contribute new neuroscientific evidence supporting this hypothesis. This paper presents a systematic review of the international literature from the past 15 years focused on visual attention in the deaf population. The final review included 24 articles. The function of the orienting network is found to be enhanced in deaf adults and children, primarily observed in native signers without cochlear implants, while endogenous orienting is observed only in the context of gaze cues in children, with no differences found in adults. Results regarding alerting and executive function vary depending on clinical characteristics and paradigms used. Implications for future research on visual attention in the deaf population are discussed.

## 1 Introduction

### 1.1 Background

Early auditory deprivation is recognized as a factor influencing the development of visual attention in deaf individuals (Colmenero et al., 2004; Bavelier et al., 2006; Stevens and Neville, 2006). However, existing evidence on the nature of this effect is conflicting and, crucially for the present review, unclear concerning the temporal versus spatial distribution of visual attention. Historically, research on this topic has been centered on two seemingly opposing hypotheses: the deficiency hypothesis, positing that early profound deafness leads to visual attention deficits, and the enhancement hypothesis, suggesting compensatory changes to visual attention processes (Dye and Bavelier, 2010).

According to *the deficiency hypothesis*, integrating information from different senses is essential for the normal development of attention functioning within each sensory modality. Consequently, the absence of auditory input results in underdeveloped selective attention capacities. For deaf individuals, the lack of audition impairs the development of multisensory integration, thereby impeding the typical development of visual attention skills. Put simply, while hearing people can selectively attend to a narrow visual field and still monitor the broader environment through sounds, deaf individuals must use vision to accomplish both specific tasks and monitor the broader environment (Smith et al., 1998).

This view has been primarily supported by studies examining sustained visual attention or vigilance using the Continuous Performance Test or “CPT.” For example, using the Gordon Diagnostic System (GDS), a widely used CPT, the participant is presented with digits and must respond when a “1” is followed by a “9” for around 10 min (Dye and Hauser, 2014). These studies have found consistent underperformance in CPTs among the deaf population, indicating that auditory input plays a role in organizing visual attention. These results are consistent with a deficit view of cross-modal reorganization stemming from early sensory deprivation (Mitchell and Quittner, 1996; Smith et al., 1998; Quittner et al., 2004).

Although CPTs have been widely used to assess sustained visual attention, these tasks are sensitive to certain additional cognitive factors (Parasnis et al., 2003). Specifically, CPTs require sustained attention and the ability to hold information about the target sequence in working memory, and performance is negatively affected by the inability to inhibit responses to non-target stimuli.

In contrast to the deficiency hypothesis, the *enhancement hypothesis* or *compensation view* is based on the common assumption that deficits in one sensory modality lead to heightened sensitivities in the remaining modalities (Bavelier et al., 2006). In the case of early deafness, this perspective posits that the visual system is reorganized to compensate for the lack of auditory input. Consequently, visual skills assume the functional roles previously performed by audition in the typically developing child, such as monitoring the environment or discriminating temporally complex stimuli (Bottari et al., 2014; Benetti et al., 2017; Bola et al., 2017; Seymour et al., 2017).

The enhancement or compensation hypothesis has primarily received support from studies measuring the allocation of attention across space. The results of these studies suggest that in deaf individuals, there is a spatial redistribution of visual attention toward the periphery, allowing them to better monitor their peripheral environment based on visual rather than auditory cues (Loke and Song, 1991; Sladen et al., 2005). For example, deaf individuals can be faster than hearing controls in detecting the onset of peripheral visual targets (Chen et al., 2006; Bottari et al., 2010; Codina et al., 2011, 2017) or in discriminating the direction of visual motion with attention to peripheral locations (Neville and Lawson, 1987; Bavelier et al., 2001).

This redistribution of visual attention can alter the trade-off in the responses of deaf people to the periphery versus the centre. Specifically, in situations where central and peripheral static stimuli compete for selective attention resources, deaf participants are more likely to orient visual attention toward peripheral than central locations (Sladen et al., 2005; Chen et al., 2006). Consistent with these findings, Proksch and Bavelier (2002) observed that deaf individuals are more distracted by irrelevant peripheral information, whereas hearing individuals are more distracted by irrelevant central information. However, while deaf individuals have been shown to possess a field of view that extends further toward the periphery than hearing controls (Sladen et al., 2005), no differences between deaf individuals and hearing controls have been documented when processing targets presented toward the centre of the visual field (Neville and Lawson, 1987; Loke and Song, 1991).

In an initial review conducted by Tharpe et al. (2008) to examine evidence-based literature on visual attention and deafness, various paradigms were explored, including the CPT, the letter cancellation task, and conflict tasks. No conclusive evidence was found to support general enhancement or deficits in visual attention or enhanced fundamental visual sensory abilities (Tharpe et al., 2002). Rather, the authors propose that the variability in performance across these paradigms could be explained by the extensive allocation of attentional resources across the visual field, driven by increased monitoring demands. This hypothesis explains why deaf individuals tend to show poorer performance on tasks requiring sustained attention to central stimuli over time compared to those involving the detection of peripheral stimuli. This idea has been supported by results found using a modified flanker paradigm incorporating several degrees of distance between distractor and target (Sladen et al., 2005).

Functional brain studies have also revealed significant differences between deaf and hearing individuals that support the compensation view. These differences are related to alterations in the visual areas and the activation of visual and attention-related brain networks. For instance, Bavelier et al. (2001) found that the absence of auditory input and sign language use in the deaf population was associated with greater activation of visual cortex areas when processing peripheral and moving stimuli. Furthermore, Mayberry et al. (2011) reported that deaf individuals exhibited greater activation of visual and attention-related brain networks during peripheral visual tasks.

An area of the cortex that has been extensively studied in the context of deafness is the middle temporal (MT) or medial superior temporal (MST) area. MT/MST areas play a key role in detecting and analyzing movement and activity in these areas is modulated by attentional processes (O’Craven et al., 1997). When observing unattended moving stimuli, both deaf and hearing participants show similar recruitment of the MT/MST cortex. However, when required to attend to peripheral movement and ignore concurrent central motion, enhanced recruitment of the MT/MST is observed in deaf individuals relative to hearing controls (Bavelier et al., 2001; Fine et al., 2005). This pattern echoes a general trend in the literature, where the most significant population differences have been reported for motion stimuli in the visual periphery under conditions that engage selective attention, such as when the location or time of arrival of the stimulus is unknown or when the stimulus must be selected from distractors (Bavelier et al., 2006). These findings suggest that deafness is associated with alterations in visual attention, resulting in changes in the recruitment of brain networks involved in the processing of visual information.

These apparently contradictory hypotheses highlight the necessity of organising previous research within a recognized model of attention. This review aims to respond to this need by systematically analysing the tasks employed to measure various aspects of attention in each study.

### 1.2 The integrative hypothesis

The contradictory results mentioned previously prompted an integrative review published by Dye and Bavelier (2010). These authors proposed that while the *deficiency hypothesis* and *enhancement hypothesis* may appear to be mutually exclusive, the conflicting evidence concerning the impact of deafness on visual attention could arise from measuring different aspects of visual attention. Consequently, the deficit view is predominantly supported by studies focused on the allocation of attention over time, whereas the compensation view is backed by studies measuring the allocation of attention across space. Therefore, when considering different aspects of visual attention, a striking pattern of attentional enhancements and deficits emerges as a consequence of early deafness.

In addition, these two perspectives consider groups of different ages and backgrounds. Individuals in the deaf and hard of hearing population are quite diverse regarding their preferred mode of communication (sign language versus oral language), the age of acquisition of their native language, the hearing status of their parents, the aetiology of hearing loss (e.g., genetic, infection), and the implantation of cochlear implants [CI—a small electronic device that is surgically implanted into the inner ear to help provide a sense of sound to individuals with severe to profound hearing loss (Wilson and Dorman, 2008)]. Most of the research suggesting that deaf children have problems with visual attention has focused on deaf children learning spoken language, examining changes in sustained visual attention after restoration of auditory input through a CI (Mitchell and Quittner, 1996; Smith et al., 1998; Quittner et al., 2004). In contrast, studies suggesting that the visual system compensates for the lack of auditory input by enhancing the monitoring of the peripheral visual field have primarily involved deaf adults. Specifically, these studies have focused on culturally deaf individuals born to Deaf parents, acquiring American Sign Language (ASL) as their first language and lacking CI. This group is compared to those who received oral speech therapy and have CI (Bavelier et al., 2006; Dye et al., 2009).

Dye and Bavelier (2010) suggested that the deficiency and compensatory views were not necessarily contradictory but complementary in explaining the cross-modal reorganization of visual attention after early deafness. They propose an *integrative view* in which early auditory deprivation does not have an overall positive or negative impact on visual attention, but rather, selected aspects of visual attention are modified in various ways throughout the developmental trajectory.

However, this division of visual attention in temporal and spatial aspects is very broad, and the paradigms used to test these hypotheses have certain shortcomings. Studies examining the impact on temporal attention used measures from the *Rapid Serial Visual Presentation Paradigms* and the *Attentional Blink;* however, consistent results were not observed across different experiments (Dye and Bavelier, 2010; Dye, 2014; Thakur et al., 2019). Concerning spatial attention, the Useful Field Of View (UFOV) task has been employed. However, this complex dual task requires following two instructions — to both detect and locate a target while ignoring several distractors. Consequently, working memory, inhibition, orienting, and divided attention can all be deployed in this task, giving rise to what is referred to as the *task impurity problem* (Miyake et al., 2000).

### 1.3 The attention networks model

Understanding the potential deficits and enhancements in visual attention among deaf individuals requires recognizing that visual attention is not a unitary entity. From this perspective, based on behavioral and neuroscientific studies, Posner and colleagues have suggested a model that divides the human attentional system into three functionally and anatomically independent networks responsible for alerting, orienting, and executive attention (Fan et al., 2002, 2005; Posner and Rothbart, 2007; Petersen and Posner, 2012). As already mentioned, previous hypotheses suggest that various aspects of visual attention can be affected differently in deaf individuals due to compensatory changes. The attentional networks model offers a framework to measure these different changes by separating attention into several functions.

The *alerting network* is responsible for achieving and maintaining a state of elevated sensitivity to incoming information. Alertness can be further subdivided into tonic and phasic alertness (for a review, see Sturm and Willmes, 2001). Tonic alertness (also called vigilance or sustained attention) is a state of general wakefulness or vigilance and refers to the ability to sustain attention over a period of time. Phasic alertness is a more transient alert state, modulated by a warning that precedes a target stimulus and prepares the individual for a fast reaction. Performance within this network has been measured using tasks where the appearance of the target is preceded by an anticipatory alerting cue, provoking a phasic change in alertness. This transition involves a shift from a resting state to a prepared state, ready to detect and respond to an expected event (Marrocco and Davidson, 1998; Beane and Marrocco, 2004). Tonic alertness, on the other hand, is typically evaluated through lengthy and repetitive tasks requiring participants to identify and respond to infrequently occurring targets, the most frequent example being CPTs (Petersen and Posner, 2012).

The *orienting network* is responsible for the movement of attention throughout space, allowing the selection of specific information from numerous sensory inputs. In this regard, *orienting* can be reflexive (*exogenous*), such as when a sudden target event draws attention to its location, or it can be voluntary (*endogenous*), such as when a person searches the visual field looking for a target (Jonides, 1981). Although *overt orienting* is often associated with head or eye movements toward the target, it can also enhance target processing by covertly orienting attention (Posner, 1980, 2016). Spatial orientation has traditionally been studied with tasks based on the “spatial orienting paradigm” or “cost and benefits paradigm.” In these tasks, the participants are presented with a fixation point and placeholders (the location where the target appears) at both sides of a fixation point. Following the onset of the fixation point, an attentional cue is presented, followed by the target to which participants must respond. Trials are categorized as cued/valid if the target appears at cued locations, uncued/invalid when it appears opposite to the cue, or neutral when the cue appears at the centre or both locations. In typical measures of exogenous orienting, a change occurs in the placeholder location to elicit an involuntary orienting response (such as the illumination of the locations). Conversely, in measures of endogenous orienting, a central cue is presented to prompt a voluntary orienting response toward a specific location or object (Uncapher et al., 2011; Chica et al., 2014).

Finally, *the executive attention network* involves more complex mental operations to detect and resolve the conflict between expectation, stimulus, and response. While this network shares some overlap with executive functions, it specifically involves processes related to planning and executing goal-directed actions. However, executive functions are a more general domain that includes working memory, mental flexibility, conflict monitoring, and, in close association with executive attention, inhibitory control (Botvinick et al., 2001; Matsumoto and Tanaka, 2004). Assessment of the executive attention network typically involves “resolution of conflict” paradigms, which require the suppression of either processing or responding to information that elicits incorrect or inappropriate responses (Posner and DiGirolamo, 1998). Examples of such paradigms include the flanker (Fan et al., 2002), Stroop (Fan et al., 2003), or Simon tasks (Simon and Craft, 1970).

One commonly used task specifically designed to measure most of these networks is the *attention network test* (ANT), which is based on two paradigms — the flanker task and the cost and benefits paradigm. The ANT enables the evaluation of three attentional networks in children and adults: phasic alerting, exogenous orienting, and executive attention (Fan et al., 2002).

The main task is based on the flanker paradigm where the participant must press two keys indicating the direction (left or right) of a central arrow surrounded by congruent, incongruent, or neutral flankers. The difference in reaction times or accuracy between the congruent and incongruent conditions provides a measure of the executive attention network. The efficiency of the alerting network is examined by changes in performance resulting from a warning signal preceding the target, compared to trials without any previous cue. The efficiency of the orienting network is measured by comparing the performance benefits associated with a spatial cue predicting the location of the stimulus array (above or below fixation) with a central cue.

The integrative hypothesis proposed by Dye and Bavelier (2010) predicts that the strengths and weaknesses in visual attention resulting from early auditory deprivation are also linked to the abilities of orienting, alerting, and executive functions within the visual attention networks model developed by Petersen and Posner (2012). Consequently, it is important to identify the tasks used to measure attention in deaf individuals and their possible interpretation according to the attention networks model. Understanding the weaknesses and strengths of visual attention networks related to early auditory deprivation aids in characterizing the developmental trajectory of these attentional functions during middle childhood (from 6 to 12 years old) since this is an important developmental stage for visual attention (Rueda et al., 2004) and marks the beginning of formal schooling.

### 1.4 Objectives

To our knowledge, no systematic review has included evidence regarding the integrative hypothesis proposed by Dye and Bavelier (2010). Furthermore, since the publication of the 2008 review by Tharpe and colleagues, no comprehensive review has been conducted to gather research findings enabling the identification of visual attention functions that could be diminished or enhanced in individuals with early auditory deprivation.

We conducted a systematic review of studies published between 2008 and 2023 focusing on deaf populations (from middle childhood through adulthood). The objective was to analyse investigations exploring one or more visual attention functions described in the attentional networks model. More specifically, our systematic review aims to:

1. Determine the most frequently studied functions of alerting, orienting, and executive attention in deaf individuals, along with the task paradigms employed to investigate such functions.
2. Identify the main strengths and impairments observed in the functioning of attentional networks in deaf adults and explore whether differences are found depending on the use of different communication systems, cochlear implants, and age of cochlear implant acquisition.
3. Examine the key developmental changes observed in the functioning of attention networks in deaf children during middle childhood (ages 6–12) and identify the main differences compared to typical hearing children of the same age.

## 2 Methods

### 2.1 Search strategy

We conducted a search on October 9th, 2023, of the peer-reviewed literature published in English between 2008 and 2023. The search was carried out on the *Web of Science, Medline, Scielo, and Psycinfo* databases, focusing on experimental studies of deaf populations aged 6–50 years. Using performance tasks to measure visual attention. The search utilized specific terms with relevant connectors to target visual attention measures and the population of interest. The search terms included: (deaf* OR “auditory deprivation” OR “hearing impairment”) AND (“orient*” OR “alert*” OR “spatial attention” OR “attention network” OR “visual selective attention” OR “visual attention” OR “sustained attention” OR “altered attention” OR “divided attention” OR “visuospatial attention” OR “executive attention”). Data extraction adhered to the recommendations provided by the Cochrane group (Higgins and Green, 2011) and the Preferred Reporting Items for Systematic Review and Meta-Analyses protocol (PRISMA; Moher et al., 2009).

### 2.2 Selection criteria

We use the PICOS strategy to define inclusion criteria (Participants, Intervention, Comparisons, Results, and Studies). This review includes studies with the following characteristics: (P) participants without a psychiatric history and typical neurodevelopment with mild, severe, or profound bilateral deafness aged between 6 and 50 year; (I) measures of some of the specific functions of the attention networks, including alerting orienting and executive attention. No specific intervention is considered in this review; (C) Transversal studies comparing performance between the deaf and typical hearing population, studies that compare the deaf population across different clinical variables such as CI and system of communication, and longitudinal studies within the deaf population assessing the development of visual attention; (O) studies are included where at least some of the attention networks can be separately measured through performance-based tasks based on the previously mentioned paradigms; and (S) Single case studies, doctoral theses, conference presentations, and papers without peer review are excluded.

### 2.3 Data extraction and quality evaluation

**Figure 1:**
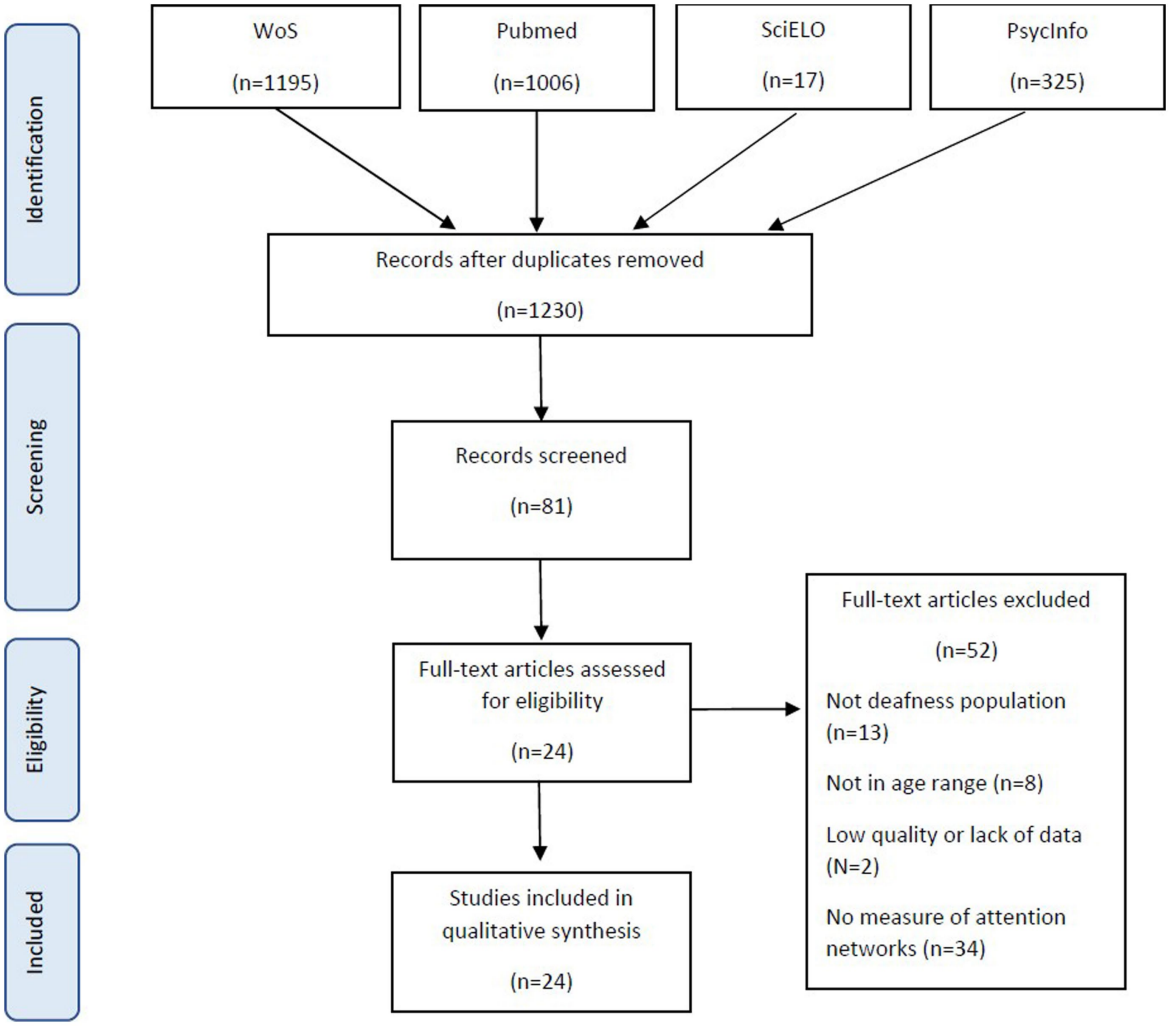
Flow chart of the identification, screening, eligibility, and selection of studies.

The initial search yielded 2,603 articles. After excluding duplicates between databases, 1,349 articles were removed. After applying the exclusion criteria, the studies were filtered by title and abstract, resulting in 86 remaining papers by the first author. The full texts of these 86 articles were then read and analysed by all authors. Most articles were excluded due to the inclusion of populations with other deficits, non-performance-based measures, or tasks that measured other aspects of visual attention not included in the attention networks model. In total, 24 articles met our inclusion criteria in agreement with all authors (see Figure 1).

Based on our research objective, the articles were classified according to the age of the participants: individuals aged 18 to 60 were categorized as adults, while those aged 6 to 12 were considered children. After some deliberation among the authors, studies involving participants up to 14 years old were included in the children category, along with a study by Kronenberger et al. (2013) which encompassed individuals aged 7–25 years. Significant statistical differences between deaf or hard of hearing and fully hearing individuals in measures assessing attention network efficiency were used as an indication of specific outcomes for each study.

The risk of bias was assessed for all articles using the Newcastle-Ottawa scale (NOS; Wells et al., 2021, adapted from Herzog et al., 2013) to evaluate the quality of the studies. In this version, the quality scores were based on the selection of sample, comparability between groups, and the measurement of results. For cross-sectional studies, a maximum score of 10 can be obtained, with a score above 6 considered a satisfactory methodology score (Orton et al., 2014). In this systematic review, none of the studies included in the final analysis scored <7 (see Table 1).

**Table 1:** Risk of bias scores adapted from the Newcastle-Ottawa scale.

Source	Sample representation	Sample size	Availability and replicability of evaluations	Anamnestic data related to deafness	Sociodemographic comparisons and control	Assessment of outcome	Statistical tests	Score/11
Yucel and Derim (2008)	*		**	*	**	*	*	8
Chen et al. (2010)	*		**	*	*	*	*	7
Xingjuan et al. (2011)	*		**	*	**	*	*	8
Bottari et al. (2012)	*		**	**	*	*	*	8
Hauthal et al. (2012)	*		**	*	**	*	*	8
Kronenberger et al. (2013)	*	*	**	**	**	*	*	10
Daza and Phillips-Silver (2013)	*		**	**	**	*	*	9
Dye and Hauser (2014)	*	*	**	**	*	*	*	9
Heimler et al. (2015a) ^1^	*		**	**	*	*	*	8
Heimler et al. (2015b) ^2^	*		**	**	*	*	*	8
Prasad et al. (2015)	*		**	**	*	*	*	8
Jayaraman et al. (2016)	*		**	**	*	*	*	8
Hoffman et al. (2018)	*	*	**	**	**	*		9
Pavani et al. (2019)	*		**	**	**	*	*	9
Bharadwaj et al. (2020)	*		**	**	*	*	*	8
Holmer et al. (2020)	*		**	*	**	*	*	8
Brazão et al. (2021)	*	*	**	*	*	*	*	8
Daza González et al. (2021)	*		**	**	**	*	*	9
Bonmassar et al. (2021)	*	*	**	**	**	*	*	10
Li et al. (2022)	*	*	**	*	*	*	*	8
Dye and Terhune-Cotter (2023)	*	*	**	**	**	*	*	10
Merchán et al. (2022)	*	*	**	**	**	*	*	10
Prasad et al. (2022)	*	*	**	*	*	*	*	8

## 3 Results

### 3.1 Frequency of studies and tasks used

The initial objective of the study was to determine the most frequently studied aspects of attention. Of the 24 included studies, 23 adopted a cross-sectional experimental design and one was a longitudinal study. Additionally, 15 studies focused solely on adult samples, eight studies exclusively involved children, and one study included both adult and child participants. Not all studies investigated a single attention network (see Table 2).

**Table 2:** Studies included in the review by group of age sample and function measured.

	Alerting		Orienting		Executive attention
	Tonic	Phasic	Exogenous	Endogenous	
Children	Dye and Hauser (2014), Hoffman et al. (2018), Yucel and Derim (2008), and *Dye and Terhune-Cotter (2023)	*Daza and Phillips-Silver (2013)	*Daza and Phillips-Silver (2013)	Pavani et al. (2019)	*Daza and Phillips-Silver (2013), Dye and Hauser (2014), Daza González et al. (2021), and Merchán et al. (2022)
Adults	*Bharadwaj et al. (2020)	Prasad et al. (2022)	Bottari et al. (2012), Brazão et al. (2021), Heimler et al. (2015a)^1^, Jayaraman et al. (2016), Prasad et al. (2015), Prasad et al. (2022), Xingjuan et al. (2011), and Li et al. (2022)	Heimler et al. (2015a,b)^1,2^, Bonmassar et al. (2021) (18–49), and Li et al. (2022) (30–45)	Chen et al. (2010), Hauthal et al. (2012), and Holmer et al. (2020)
Children and adults	Kronenberger et al. (2013)				

^*^Studies without full hearing sample.

#### 3.1.1 The alerting network

The alerting network was studied in 8 of 24 articles. Among these studies, five exclusively involved children aged between 6 and 12 years, two studies focused on adults aged between 19 and 57, and one study used a mixed sample of adults and children aged between 7 and 25. Except for two of the 11 studies (Daza and Phillips-Silver, 2013; Bharadwaj et al., 2020), the rest compared the deaf group with their hearing peers. Six of the 11 studied the tonic alerting network using CPTs, while one studied phasic alerting using the ANT (see Table 3).

**Table 3:** Paradigms used in the studies included in the review.

Attention network	Paradigm/task/test	Studies
*Alerting*		
Tonic	Continuos perfermance tasks (CPTs)	Bharadwaj et al. (2020), Dye and Hauser (2014), Hoffman et al. (2018), Kronenberger et al. (2013), Yucel and Derim (2008), and Dye and Terhune-Cotter (2023)
Phasic	Alerting cues	Daza and Phillips-Silver (2013) and Prasad et al. (2022)
*Orienting*		
Exogenous	Spatial orienting paradigm	Xingjuan et al. (2011), Bottari et al. (2012), Daza and Phillips-Silver (2013), Prasad et al. (2015), Jayaraman et al. (2016), Prasad et al. (2022), Brazão et al. (2021), and Li et al. (2022)
	Attention network test (ANT-child)	Daza and Phillips-Silver (2013)
	Visual search paradigm	Heimler et al. (2015a) ^1^
	Spatial orienting paradigm	Heimler et al. (2015b)^2^, Pavani et al. (2019), Bonmassar et al. (2021), and Li et al. (2022)
Endogenous	Visual search paradigm	Heimler et al. (2015a) ^1^
Executive attention	Attention network test (ANT-child)	Daza and Phillips-Silver (2013), Daza González et al. (2021), and Merchán et al. (2022)
	Conflict tasks	Chen et al. (2010), Hauthal et al. (2012), and Holmer et al. (2020)

As mentioned above, CPTs are frequently used to measure visual attention in deaf individuals. Depending on the paradigm used, several interpretations are possible regarding the specific function measured. Following the previously described example of the GDS, commission errors due to responding to “9” when no “1” appeared are considered impulsive, lack of response or omission errors are considered distraction/inattention, and the most commonly used “*d*’” combines commission and omission errors to obtain a measure of sensitivity and is considered to show vigilance, which is why it has been classified as a tonic alerting measure (Baijot et al., 2013).

#### 3.1.2 The orienting network

The orienting network was studied in 12 of the 24 articles. Only two studies focused on children, one involving a sample aged between 6 and 14 years and another involving both children and adults aged between 10 and 58. The majority of studies (seven out of 12) were conducted exclusively with adults aged between 18 and 57. In eight out of 12 articles, exogenous orienting was studied, using spatial orienting paradigms, including the ANT. Four articles investigated endogenous orienting using spatial orienting paradigms. A visual search paradigm designed by Heimler et al. (2015a) allows for obtaining a measure of exogenous and endogenous orienting and was included in both categories (see Table 3).

As mentioned previously, the orienting paradigms facilitate the measurement of exogenous and endogenous orienting by manipulating cues before the appearance of targets. These paradigms provide various measures of the orienting process, the most common being the facilitation of a valid cue toward the target. Additionally, they can be used to measure the disengagement of attention following an invalid cue. In cases where eye movements are considered, overt orienting of attention is measured instead of covert attention. Four of the nine studies focusing on exogenous attention with orienting paradigms measured saccadic eye movements (overt attention), while the remaining five used only manual responses (covert attention).Heimler et al. (2015a) designed a visual search paradigm in which participants must search for a target (tilted line) among a visual field full of similar distractors (straight lines) while ignoring a salient distractor (line tilted opposite direction). The salience of the target and distractor was manipulated trial by trial by changing their colors. This approach was driven by the idea that the salient stimulus attracts exogenous attention while the target requires an endogenous search across the visual field. Through this method, they were able to obtain a measure of endogenous orienting and exogenous orienting.

#### 3.1.3 The executive attention network

**Figure 2:**
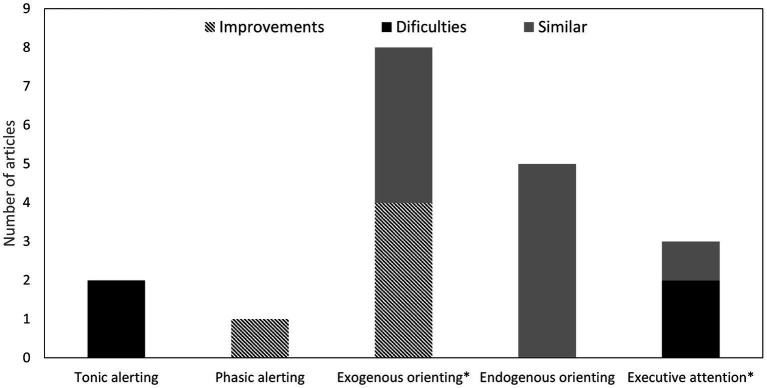
Number of studies with deaf children in each attention network function and the general findings in comparison to full hearing children. *Deficits in executive attention found with younger children [2, 74].

**Figure 3:**
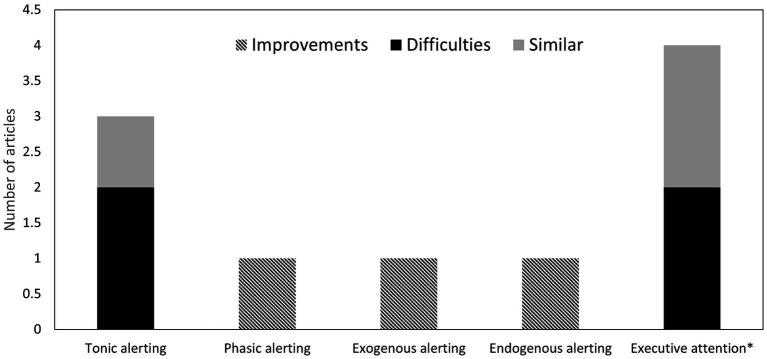
Number of studies with deaf adults in each attention network function and the general findings in comparison to full hearing adults. *Enhancements found in overt orienting but not in covert orienting and deficits with executive attention found only during specific conditions.

The executive attention network was studied in 7 of 24 articles. Four studies involved a sample of children aged between 6 and 13 (see Figure 2), while three focused exclusively on adults aged between 18 and 58 (see Figure 3). Three of the 7 used conflict tasks with several modifications, three used the ANT, and the remainder employed the modified CPT developed by Dye and Hauser (2014) (see Table 3).

Regarding measures of executive attention, paradigms classified as conflict tasks were included. These tasks require participants to ignore distractors while attending to a central target. Notably, the study conducted by Dye and Hauser (2014) used a CPT but compared the execution of a CPT with and without distractors in the same sample, thereby measuring executive attention.

### 3.2 Comparisons between deaf and hearing adults

Our second objective was to compile the differences found between deaf and full hearing adults. We note that all studies involving adults employed a cross-sectional design (see Figure 2). The two studies that measured tonic alerting in deaf adults revealed poorer performance compared to hearing peers when using CPTs as a measure (Kronenberger et al., 2013; Bharadwaj et al., 2020). Specifically, Kronenberger et al. (2013) used the *Test of Variables of Attention* (TOVA), indicating poorer performance by deaf individuals across all measures. Similarly, Bharadwaj et al. (2020) used *the Integrated Visual and Auditory Continuous Performance Test* (IVA plus CPT), demonstrating that deaf individuals commit more omission errors and have slower reaction times.

The only study that measured phasic alerting with alerting cues found an advantage in deaf adults (Prasad et al., 2022). Deaf adults also seem to have advantages in covert exogenous orienting (Xingjuan et al., 2011; Brazão et al., 2021; Li et al., 2022; Prasad et al., 2022). However, studies that measured overt attention with saccadic movement found no facilitation of exogenous orienting with this measure (Bottari et al., 2012; Prasad et al., 2015; Heimler et al., 2015a; Jayaraman et al., 2016). The five studies that measured endogenous orienting found no differences between deaf and full-hearing adults (Bottari et al., 2008; Heimler et al., 2015a,b; Bonmassar et al., 2021; Li et al., 2022).

Regarding executive attention, no differences were found between deaf adults and their hearing peers in a typical flanker task (Holmer et al., 2020). Chen et al. (2010) used a paradigm with three conditions: congruency, the distance of the distractor (central or peripheral), and screen proximity (typical computer screen or projected onto a wall) and found that deaf adults showed greater interference from peripheral distractors compared to central cues. This effect was reversed when the display was projected onto a wall. Hauthal et al. (2012) designed a paradigm where participants had to discern the gender of a central target while faces appeared as distractors at the flanks. The faces could either match or differ in gender from the target, creating interference. The study revealed that with a high volume of distractors, adult deaf signers without CI still showed interference effects while hearing adults did not.

Regarding our secondary objective, to explore any differences observed among deaf individuals in relation to variables concerning hearing loss history, device use and mode of communication, very few studies examined adults with CI (see Table 4), possibly due to the relative novelty of the technology (Wilson and Dorman, 2008). The few studies that included adults with CI did not find any effect of implantation in tonic alerting (Kronenberger et al., 2013; Bharadwaj et al., 2020). The rest of the findings will be discussed below along with the results of studies in children.

**Table 4:** Basic deaf related variables regarding auditory access and system of communication.

Source	Age	Cochlear implantation	Age of acquisition of CI	Hearing condition of parents	Use of sign language
Yucel and Derim (2008)	6–11	All deaf sample with CI	39.13 (10.3) and 69.66 (12.7) months	n/a	Verbal therapy required
Chen et al. (2010)	18–24	n/a		n/a	First language from birth
Xingjuan et al. (2011)	18–24	n/a		n/a	Native sign language users
Bottari et al. (2012)	24–55	No		4 of 13 learned sign language from deaf parents	All preferred sign language
Hauthal et al. (2012)	28–58	n/a		n/a	All fluent in sign language
Daza and Phillips-Silver (2013)	6–13	14 with CI 14 without CI	1–6 years of usage	n/a	14 preferred oral language 14 preferred sign language
Kronenberger et al. (2013)	7–25	All deaf sample with CI	38 (19.3) months	n/a	Mostly oral speakers
Dye and Hauser (2014)	6–12	All deaf sample with CI		Born from deaf parents	Bilingual, better ASL proficiency
Heimler et al. (2015a) ^1^	20–44	“Hearing aids” 15/22	n/a	n/a	Measured, mostly high proficiency
Heimler et al. (2015b) ^2^	20–44	n/a	n/a	6 out of 19 born from deaf parent	Measured, mostly high proficiency
Prasad et al. (2015)	19–33	N/a		All born from deaf parents	All knew sign language
Jayaraman et al. (2016)	18–33	n/a		All born from hearing parents	All highly proficient in sign language
Hoffman et al. (2018)	6–8	All deaf sample with CI	1.92 (0.89) years	n/a	Commitment to oral education
Pavani et al. (2019)	7–14	No		13 of 16 at least one deaf parent	All deaf used and knew sign language
Bharadwaj et al. (2020)	24–57	13 of 15	38.66 (11.22) months	n/a	Oral speakers but 6 of 15 knowledge of sign language
Holmer et al. (2020).	19–48	n/a		5 of 16 born from deaf parents	Sign language as primary language
Daza González et al. (2021)	9–10	9 out of 17 had CI	4.0 (1.8) years	All born from hearing parents	5 out of 17 bilingual and 2 exclusive sign language
Brazão et al. (2021)	22–53	No		2 out of 11 born from deaf parent	High sing language proficiency
Bonmassar et al. (2021)	18–49	No		n/a	Users of sign language mostly native
Prasad et al. (2022)	22–44	n/a		All born from hearing parents	High sign language proficiency
Merchán et al. (2022)	7–10	19 with CI 16 without CI	5 before 2 y/o 9 between 3 and 5 y/o 5 after 5 y/o	n/a	12 preferred oral language 23 preferred oral language with sign language
Dye and Terhune-Cotter (2023)	7–13	4 out of 88		75 from deaf family 10 from hearing family 3 n/a	Oral and sign language bilinguals
Li et al. (2022)	30–45	n/a	3,2 years	n/a	37 of 42 familiar with sign language (above 3 out of 5 in familiarity scale)

### 3.3 Development of attention networks and comparison between deaf and full hearing children

With respect to our third and final objective, we found one longitudinal study and four studies that either compared groups across different ages or treated age as a continuous independent variable. In deaf individuals, tonic alerting was observed to develop between 6 and 13 years of age (Dye and Hauser, 2014; Dye and Terhune-Cotter, 2023). With exogenous orienting, the only result found was that the fundamental operations of moving and engaging develop from 6 to 7 years of age (Daza and Phillips-Silver, 2013). Lastly, executive attention appears to develop around 8 years of age in deaf individuals (Dye and Hauser, 2014). As mentioned before, comparisons between differences between individuals with and without CIs and different systems of communication were almost exclusive to studies with children. When comparing deaf and typical hearing children, greater challenges in tonic alertness were evident in speaking deaf children with CI (Yucel and Derim, 2008; Hoffman et al., 2018), but not in deaf signers without CI (Dye and Hauser, 2014). Regarding phasic alertness, Daza and Phillips-Silver (2013) found a greater alerting effect in the ANT when comparing oral deaf children with CI and deaf signers without CI. Daza and Phillips-Silver (2013) also found faster movement and engagement in a spatial orienting paradigm when comparing deaf signers without CI to oral deaf children with CI. However, in endogenous orienting, an advantage was found in deaf children (independent of the system of communication) when a social central cue was employed (Pavani et al., 2019). When measuring executive attention with a flanker task, no differences were found between deaf children (mostly speaking with CI) and hearing children (Daza and Phillips-Silver, 2013; Daza González et al., 2021) except for Merchán et al. (2022) who observed poorer performance in deaf children. Dye and Hauser (2014), examining the effect of distractors on a central target with a focus on the difference in performance on two CPTs, found that deaf signers without CI showed poorer performance than their full hearing counterparts.

## 4 Discussion

### 4.1 Current frequency of studies and tasks used

As observed, there exist notable gaps in our understanding of the visual attention network in deaf individuals, with research focusing on different functions depending on the age of the participants. While tonic alerting has been extensively researched in both adult and youth deaf populations, primarily through CPTs, the exploration of phasic alerting remains scarce in both groups. Notably, only one study in adults has investigated phasic alerting, emerging as an unexpected result from a cost and benefit paradigm measuring orienting behavior (Prasad et al., 2022).

Similarly, concerning the orienting network, while there is a wide range of research on exogenous orienting in deaf adults, few studies have tested these differences in deaf children (Daza and Phillips-Silver, 2013). Moreover, endogenous attention has been underexplored in both age groups.

Regarding executive attention, there appears to be a more balanced interest across developmental stages, primarily through flanker tasks in children and a broader range of conflict tasks in deaf adults. This is likely due to the fact that flanker tasks have been previously studied in deaf adults prior to the scope of this review (Sladen et al., 2005; Dye et al., 2009).

From these observations, it becomes evident that there is a critical need to delve deeper into the exploration of phasic alerting and endogenous orienting of attention, particularly in deaf and hard of hearing children. This need arises from the potential existence of adaptive developmental aspects in visual attention that warrant further investigation.

### 4.2 Results of comparisons between deaf and hearing adults

The results in deaf adults seem to indicate a deficit in the tonic alerting network, which can be explained by several hypotheses. One possibility is that deaf individuals have difficulties in sustaining attention over time, possibly due to a more rapid depletion of attentional resources. To test this hypothesis, investigating how performance changes over the course of a task could provide insights into whether there is a faster decline in performance or a general difficulty in executing the task. While Hoffman et al. (2018) attempted to analyse this aspect, they focused exclusively on children, which will be discussed below. Another hypothesis emerges from the division of labor perspective, which supports the deficit view. According to this notion, the observed results may be due to the need for deaf individuals to rely on vision to simultaneously monitor their environment and focus on a specific task. This dual demand on attentional resources might limit the resources available for performing visual tasks such as the CPT (Smith et al., 1998; Quittner et al., 2004).

Normally, phasic alerting is primarily dependent on the auditory system. Therefore, in adults, it would be reasonable to expect that adaptive mechanisms could lead to a heightened state of alert generated by visual cues, as demonstrated in the experiment conducted by Prasad et al. (2022).

The overall advantage observed in spatial exogenous orienting in deaf adults appears to be attributable to covert orienting rather than overt orienting/ eye movements (Prasad et al., 2015; Brazão et al., 2021; Prasad et al., 2022). This supports the notion of an adaptive alteration in the visual attention system in deaf individuals. This adaptation enables them to monitor the environment since they are able to efficiently shift their attentional focus across the visual field towards important stimuli and also disengage from them more rapidly.

The mechanisms governing orienting of attention or eye movements have been shown to be more dependent on endogenous attention, which could explain why the differences between deaf and full hearing individuals do not extend to the results of these tasks (Zangrossi et al., 2021; Celli et al., 2022). Endogenous orienting does not differ between deaf and typical hearing adults, whether measured by visual search (Heimler et al., 2015a) or spatial orienting paradigms using central cues (Heimler et al., 2015b; Bonmassar et al., 2021; Li et al., 2022). One explanation for this result is that endogenous orienting of attention requires voluntary control of attention (top-down), while exogenous attention is an involuntary mechanism (bottom-up), as some results indicated that deaf individuals could have worse executive control, possibly explaining the lack of differences in these tasks (Li et al., 2022). However, as we have found in this review, deficits in executive control are not common in adults or native signers, contrary to the results found in orienting. Another possible explanation for this discrepancy is that this function does not show differences since it is not inherently adaptative. In contrast, the improvements observed in exogenous orienting could stem from the need to monitor environmental changes using only the visual system, without the support of the auditory system. On the other hand, attention shifts due to endogenous attention could be distracting for deaf individuals required to maintain a strong focus on hands and facial expressions during conversations.

Regarding executive function, no differences were found in a typical flanker task (Holmer et al., 2020). However, when distractors were placed in the periphery instead of the centre, deaf individuals showed poorer performance compared to their hearing peers (Chen et al., 2010). These contradictory results seem to support the hypothesis that the observed performance deficits in conflict tasks with central targets may not necessarily be due to deficits in executive attention. Instead, these findings could be due to the further allocation of attentional resources towards distractors in comparison to hearing individuals. This explanation is further supported when these results are compared to those of the UFOV tasks, where both targets and distractors are located in the periphery. In these tasks, deaf individuals tend to have an advantage (Dye et al., 2016; Samar and Berger, 2017). However, the findings of Hauthal et al. (2012) could indicate an adaptive change specifically in the processing of faces. These results suggest that the performance of signers without CI in executive attention tasks depends on the position of the target, which can be explained by the further allocation of attention towards the periphery. An alternative interpretation of these findings is that deaf adults may develop an advantage in the ventral attention network (VAN). The VAN is responsible for reflexive bottom-up attentional mechanisms and has been associated with exogenous orienting and phasic alerting This could potentially explain the observed benefits in both functions among deaf adults. In contrast, the dorsal attention network (DAN) governs voluntary or top-down attentional mechanisms and has been linked to endogenous orienting, tonic alerting, and executive functioning (Corbetta and Shulman, 2002; Rueda et al., 2023). Apart from tonic alerting, it appears that deaf adults may not experience performance deficits relative to typical hearing controls in this pathway.

### 4.3 Findings of comparisons between deaf and hearing children

In children, the differences between deaf and typical hearing individuals vary according to age, suggesting that middle childhood is an important period of development for visual attention. Our review found that deaf children show worse scores in CPTs, which is argued as being indicative of a deficit in tonic alerting. However, contrary to this notion, Hoffman et al. (2018) found no differences in performance block by block between deaf and hearing children. This suggests that tonic alerting or vigilance may not be affected, but the difference in performance is due to the division of labor, as mentioned previously. Furthermore, the poor performance during these tasks was characterized by high commission errors (Yucel and Derim, 2008; Hoffman et al., 2018), which could be interpreted as poor inhibition or impulsivity. These findings have been replicated with other paradigms that measure response inhibition, such as the Go/No Go or Simon tasks (Figueras et al., 2008; Botting et al., 2017; Hall et al., 2018).

The results reported by Daza and Phillips-Silver (2013), indicating higher phasic alerting and faster exogenous orienting, could potentially suggest a benefit due to the lack of auditory stimulation. However, these results have not been directly compared with those of hearing children. We must also consider that the differences in endogenous attention found by Pavani et al. (2019) can only be interpreted in the context of gaze cues, since there is evidence that other (non-gaze) directional cues rely on different processes (Heimler et al., 2015b). Consequently, there is insufficient experimental data on orienting in deaf children in comparison to their hearing counterparts, which prevents us from drawing any firm conclusions in this regard.

Concerning executive attention, discrepancies between the results of Dye and Hauser (2014) and Merchán et al. (2022) and those of Daza González et al. (2021) could be due to differences in the sample, specifically in terms of age, since the latter study focused on children aged 9 to 10 years. As observed in adults, deaf children show no difference in UFOV task performance between the ages of 7 and 10 years. In fact, their performance surpasses that of their hearing peers between the ages of 11 and 17 (Dye et al., 2009; Dye and Bavelier, 2010). This discrepancy in performance might manifest as a reduced ability to ignore distractors when they are at the periphery and the target is in the centre of the visual field. Notably, this difference disappears when the target is also positioned in the periphery, supporting the hypothesis that attentional resources are allocated toward the periphery.

In general, we can conclude that, as expected, the findings have revealed improved performance of deaf individuals in tasks related to covert exogenous orienting, with limited impact on endogenous orienting in adults. However, deaf individuals show poorer execution of tasks involving tonic alertness and executive attention, except when the target is presented peripherally. These results are consistent with much of the clinical literature in deaf individuals (Barker et al., 2009) and support the integrative hypothesis suggesting a deficiency in the temporal distribution of attention and an enhancement in spatial distribution (Dye and Bavelier, 2010). Finally, from the perspective of the attention network model, our study highlights the need to further explore phasic alerting. Currently, there is a gap in research exploring differences in exogenous and endogenous orienting between deaf and full hearing children and a lack of studies investigating the endogenous orienting network in deaf adults.

### 4.4 Development of attention network functions in deaf children

Regarding our objective of characterizing the development of attentional networks in middle childhood within the deaf population, several conclusions can be drawn. However, we must consider the need for further research in this area, particularly through longitudinal studies.

Our findings indicate that tonic alertness continues to develop from ages 6 to 13 in both deaf and typical hearing children (Dye and Hauser, 2014; Dye and Terhune-Cotter, 2023). This aligns with previous research on typical hearing children using the same task, which showed a specific development ceiling at 10 years old (Betts et al., 2006). However, it appears that deaf individuals do not reach the levels observed in typical hearing adults, at least those who are not native signers (Bharadwaj et al., 2020).

The elemental operations of moving and engaging improve between the ages of 6 and 7 in deaf children (Daza and Phillips-Silver, 2013). In comparison to hearing children, our results suggest that orienting networks continue to develop during middle childhood in deaf individuals, whereas in their hearing counterparts, this development tends to plateau at around 6 years old (Rueda et al., 2004; Pozuelos et al., 2014; Federico et al., 2017). Notably, this development in deaf individuals appears to extend into adulthood, providing them with an advantage over typical hearing adults. Interestingly, when measuring electrophysiological brain activity through evoked potentials, improvements in visual attention related to saliency processing and orienting of attention have been observed as early as 3 years of age. These measures demonstrate improvement during the early years in deaf children, indicating early and differential development of these components of attention (Campbell and Sharma, 2016; de Schonen et al., 2018; Gabr et al., 2022; Corina et al., 2024).

Finally, executive attention seems to improve between 7 and 9 years of age in deaf children. Dye and Hauser (2014) found that deaf signers without CI reach the same levels of performance between 9 and 13 years old. However, Daza González et al. (2021) found no differences among children aged 9–10, while Merchán et al. (2022) observed worse performance in a sample of 7–10 years old. These findings are consistent with those reported in studies of typical hearing children, suggesting that difficulties found in this aspect of the attention network cannot be solely attributed to late development (Rueda et al., 2004; Pozuelos et al., 2014; Federico et al., 2017). When comparing deaf and hearing adults, it is plausible that deaf individuals continue to show development in exogenous orienting during early childhood, eventually achieving better performance than their hearing counterparts (Bottari et al., 2012; Prasad et al., 2015). In adulthood, deaf signers without CI reach similar levels in executive attention when central targets are present (Chen et al., 2010; Holmer et al., 2020). However, differences in tonic alertness may persist into adulthood (Parasnis et al., 2003; Kronenberger et al., 2013).

### 4.5 Effects of the communication system and the use of cochlear implants

In most studies, the use of sign language is associated with the absence of a CI. It is important to recognize the clear distinction between culturally deaf people who communicate mainly in sign language within deaf communities and those who have received CI along with speech therapy. The latter group has experienced some level of auditory input and uses a language that is less reliant on visual cues.

In adults, there are no studies on tonic alertness involving deaf signers without CI. However, age at CI implantation does not seem to have an impact on CPT performance (Kronenberger et al., 2013; Bharadwaj et al., 2020). While no differences were observed between deaf signers without CI and full hearing children (Dye and Hauser, 2014), differences have been found in oral-speaking deaf children with CI (Yucel and Derim, 2008; Hoffman et al., 2018). Additionally, Daza and Phillips-Silver (2013) found differences in tonic alertness between oral speaking deaf children with CI and deaf signers without CI in favor of the former. Dye and Terhune-Cotter (2023) found that while the English language was a strong predictor of better sustained attention, ASL proficiency was a more accurate predictor of response inhibition.

Generally speaking, these findings suggest a consistent trend toward poorer performance on tonic alerting tasks in oral speaking deaf individuals. Notably, the lack of an effect of age of implantation in adults raises the possibility that early language acquisition does not influence these outcomes. Regarding exogenous orienting, it is evident that elementary operations of orienting such as moving and engaging are enhanced in deaf signers without CI compared to oral speaking deaf individuals with CI (Daza and Phillips-Silver, 2013). Additionally, the advantage observed in executive attention towards peripheral targets in adults appears to be more prevalent among deaf signers without CI (Samar and Berger, 2017), while in children these improvements have also been found in deaf signers without CI (Dye et al., 2009).

These findings align with the main hypothesis put forward to explain differences in performance on tasks that measure different executive functions in deaf people and could also be applied to these results, that is, worse performance can be attributed to late acquisition of language (Hall et al., 2017, 2018; Merchán et al., 2022). This explanation has commonly been invoked when attempting to explain performance on executive function tasks, but as observed in this review, tonic alertness also appears to be affected. However, an adaptive form of development is evident when executive attention is directed toward the periphery in deaf signers without CI who lack auditory stimulation and have delayed acquisition of language (Dye et al., 2009; Samar and Berger, 2017). Unfortunately, there is insufficient evidence to conclusively establish the impact of these variables on phasic alertness, endogenous orienting, and executive attention.

## 5 Conclusion

In summary, there are notable gaps in the literature regarding the functions of visual attention networks, specifically in the alerting network functions in adults, phasic alerting, and both orienting networks in children. Current evidence suggests that deaf adults show poorer performance during CPTs, but this might not necessarily be attributed to deficits in tonic alerting. Phasic alerting, on the other hand, appears to confer advantages in deaf adults. Exogenous orienting shows enhancements, whereas endogenous orienting does not. Additionally, differences in executive attention are evident, particularly depending on the peripheral placement of the distractors. In children, the evidence reveals similar patterns of results, with the exception that difficulties in executive attention are observed before the ages of 9 or 10.

Regarding individual differences in language delay and the use of CI, it seems that benefits in exogenous orienting are more frequent in deaf individuals without CI and users of sign language while language abilities appear to be a good predictor of difficulties in executive attention. This understanding contributes to the growing body of knowledge in the field, emphasizing the need for further research to bridge the identified gaps and refine our comprehension of the intricate development of visual attention networks in the deaf population.

## Data availability statement

The original contributions presented in the study are included in the article/supplementary material, further inquiries can be directed to the corresponding author.

## Author contributions

NG: Writing – original draft, Methodology, Investigation. JP-S: Supervision, Conceptualization, Writing – review & editing. MD: Writing – original draft, Supervision, Writing – review & editing, Funding acquisition, Conceptualization.
